# Unrepresented and Vulnerable: A Rural/Urban Comparison of Nursing Home Decedents Without Family

**DOI:** 10.1093/geroni/igaf122.027

**Published:** 2025-12-31

**Authors:** Caroline Stephens, Rachael Alexander, Eli Iacob, Rebecca Utz, Lauren Hunt, Sei Lee, Laura Block, Katherine Ornstein

**Affiliations:** University of Utah, Salt Lake City, Utah, United States; University of Utah, Salt Lake City, Utah, United States; University of Utah, Salt Lake City, Utah, United States; University of Utah, Salt Lake City, Utah, United States; University of California, San Francisco, San Francisco, California, United States; University of California, San Francisco, San Francisco, California, United States; University of Wisconsin-Madison, Madison, Wisconsin, United States; Johns Hopkins University, Baltimore, Maryland, United States

## Abstract

Families often play a critical role in end-of-life (EOL) care for nursing home (NH) residents, yet one in five older adults who die in a NH do not have available family living in the same state at the time of death. These potentially ‘unrepresented’ NH residents are highly vulnerable to poor EOL care, yet little is known about how rurality affects this population, with rurality also contributing to EOL care inequities. Given these individuals are typically unable to participate in survey or intervention-based research studies, their needs remain largely unknown and unmet. Using the only population-based, family-linked NH dataset in the U.S. - Utah Caregiving Population Science (Utah C-PopS) - we conducted a retrospective cohort study of 9,318 NH residents identified as not having any available family (spouses, children, siblings, parents) at the time of death and compared sociodemographic and death characteristics by rural vs urban NH status. Compared to those who died in an urban NH (n = 6,960), those who died in a rural NH (n = 2,358) were more likely to be male (38.5% vs 33.7%), White Non-Hispanic (93.4% vs 87.6%), widowed (61.0% vs 59.0%) with less than a high school education (45.9% vs 39.4%). Unrepresented rural NH residents were more likely to have multiple comorbidities (18.2% vs 15.9%) and more hospitalizations in the last 30 days of life (27.7% vs 22.9%), compared to urban NH residents. Findings provide a better understanding of rural/urban differences in EOL care for vulnerable ‘unrepresented’ NH residents and advance research on disparities in EOL care.

